# Diseases of the musculoskeletal system and connective tissue in relation to temporomandibular disorders—A SWEREG-TMD nationwide case-control study

**DOI:** 10.1371/journal.pone.0275930

**Published:** 2022-10-12

**Authors:** Adrian Salinas Fredricson, Aron Naimi-Akbar, Johanna Adami, Bodil Lund, Annika Rosén, Britt Hedenberg-Magnusson, Lars Fredriksson, Carina Krüger Weiner

**Affiliations:** 1 Department of Oral and Maxillofacial Surgery, Public Dental Services, Folktandvården Stockholm, Eastmaninstitutet, Stockholm, Sweden; 2 Department of Dental Medicine, Division of Oral Diagnostics and Rehabilitation, Karolinska Institutet, Stockholm, Sweden; 3 Health Technology Assessment-Odontology (HTA-O), Malmö University, Malmö, Sweden; 4 Sophiahemmet University, Stockholm, Sweden; 5 Medical Unit for Reconstructive Plastic- and Craniofacial Surgery, Karolinska University Hospital, Stockholm, Sweden; 6 Department of Clinical Dentistry, Division of Oral and Maxillofacial Surgery, University of Bergen, Bergen, Norway; 7 Department of Oral and Maxillofacial Surgery, Haukeland University Hospital, Bergen, Norway; 8 Department of Orofacial Pain and Jaw Function, Public Dental Services, Folktandvården Stockholm, Eastmaninstitutet, Stockholm, Sweden; Thamar University, Faculty of Dentistry, YEMEN

## Abstract

**Introduction:**

Temporomandibular disorders (TMD) are comprised by a heterogenous group of diagnoses with multifaceted and complex etiologies. Although diseases of the musculoskeletal system and connective tissue (MSD) have been reported as risk factors for developing TMD, no nationwide population-based registry studies have been conducted to investigate this possible link. The aim of this study was to investigate the association between MSD and TMD in a population-based sample using Swedish registry data, and to further investigate the difference in such association between patients diagnosed with TMD in a hospital setting and patients surgically treated for the condition.

**Materials and methods:**

Population based case-control study using Swedish nationwide registry data. Data was collected between 1998 and 2016 from 33 315 incident cases and 333 122 controls aged ≥18, matched for sex, age, and living area. Cases were stratified into non-surgical (NS), surgically treated once (ST1) and surgically treated twice or more (ST2). Information on MSD exposure (ICD-10 M00-M99) was collected between 1964 and 2016. Odds ratios were calculated using conditional logistic regression, adjusted for country of birth, educational level, living area, and mental health comorbidity.

**Results:**

A significant association between MSD and the development of TMD was found for all diagnostic categories: arthropathies (OR 2.0, CI 1.9–2.0); systemic connective tissue disorders (OR 2.3, CI 2.1–2.4); dorsopathies (OR 2.2, CI 2.1–2.2); soft tissue disorders (OR 2.2, CI 2.2–2.3); osteopathies and chondropathies (OR 1.7, CI 1.6–1.8); and other disorders of the musculoskeletal system and connective tissue (OR 1.9, CI 1.8–2.1). The associations were generally much stronger for TMD requiring surgical treatment. The diagnostic group with the strongest association was inflammatory polyarthropathies, M05-M14 (OR 11.7, CI 8.6–15.9), which was seen in the ST2 group.

**Conclusions:**

Patients with MSD diagnoses have a higher probability of being diagnosed with TMD, in comparison to individuals without MSD. This association is even stronger for TMD that requires surgery. The results are in line with earlier findings, but present new population-based evidence of a possible causal relationship between MSD and TMD, even after adjusting for known confounders. Both dentists and physicians should be aware of this association and be wary of early signs of painful TMD among patients with MSD, to make early referral and timely conservative treatment possible.

## Introduction

Temporomandibular disorder (TMD) is an umbrella term for multiple diagnoses that affect the orofacial muscles and/or the temporomandibular joint (TMJ). Painful TMD affects around 10% of the population and is the second most common cause of pain in the orofacial area, and the second most common musculoskeletal condition causing disability and pain, after chronic back pain [1–3]. TMD is therefore expected to have a substantial effect on health-related societal costs, as shown in a recent study from the Swedish National Registry Studies for Surgically Treated TMD (SWEREG-TMD), where TMD patients were found to be two to three times more reliant on sick-leave and disability pension than the general population [4]. The etiology of temporomandibular disorder (TMD) is a multifaceted arrangement of biological, psychological, sleep quality and parafunctional factors, and the onset of TMD pain is believed to be a result of both premorbid conditions as well as bio-psychosocial changes over time [5, 6]. Musculoskeletal and connective tissue diseases (MSD) such as fibromyalgia and rheumatoid arthritis (RA) are strongly associated with the development of TMD, as well as endocrine risk factors, differences in anatomy, trauma, genetics, hormonal factors, and pain comorbidity [1, 7, 8].

TMD can be divided into three types of disorders based on preferred treatment options: myofascial pain and dysfunction, TMJ functional derangement, and TMJ degenerative/inflammatory joint disease [9]. The most common type of disorder is myofascial pain and dysfunction, which in Sweden is treated conservatively within the dental care system [10, 11]. In some cases, TMJ functional derangement and degenerative/inflammatory disease can be treated conservatively initially, with a re-evaluation of surgical treatment indications if conservative treatment fails. However, some conditions do not allow for conservative treatment [9]. Nevertheless, only about 1% of TMD patients are expected to be referred to an oral and maxillofacial surgeon for surgical treatment, which in Sweden is conducted within the health care system and therefore registered in the national patient registries [12]. In some cases, patients can seek medical care for their TMD, most likely due to acute onset of pain or due to symptoms located in the orofacial area without obvious connection to the teeth. These patients diagnosed within the health care system but not subsequently surgically treated can be assumed to receive conservative treatment within the dental care system.

The Orofacial Pain Prospective Evaluation and Risk Assessment Study (OPPERA) early concluded that most previous epidemiological research on TMD had been cross-sectional and many times reliant on convenience samples. As many risk factors have been implied, including bone and connective tissue disorders, OPPERA suggested that more prospective studies were warranted to establish causal pathways [13]. The OPPERA-studies has provided unique prospective and longitudinal data and research on the north American TMD population, however European or Nordic counterparts, to our knowledge, do not exist. The unique Swedish national registries hold high standards with prospectively collected data and with the SWEREG-TMD a unique opportunity is presented to investigate the association of MSD in relation to TMD, in particular surgically treated TMD. This study aims to further investigate the association of MSD and the development of TMD, and what impact such premorbid conditions have on the probabilities of becoming a patient diagnosed with TMD in a hospital setting, or surgically treated for the condition.

## Materials and methods

### Study design and registries

This case-control population study used data from the following nationwide registries:

1. The National Patient Registry (NPR), introduced in 1964, was used to identify incident cases with TMD and to collect exposure data for the cases and the controls [14]. The NPR registry, part of the National Board of Health and Welfare (NBHW), collects data on 100% of in-patient care and 80–100% of out-patient care visits. The registry includes ICD codes for primary diagnosis as well as treatment codes. The NPR was also used to collect data on prior diagnoses on mental health comorbidity.
2. The Total Population Registry (TPR), introduced in 1968, includes all Swedish citizens. The TPR was used to identify the controls [15].
3. The Longitudinal Integrated Database for Health Insurance and Labor Market Studies (LISA) was used to collect sociodemographic covariates [16]. The LISA registry covers all Swedish citizens aged ≥16 years and collects data on educational level, income, occupation, civil status, family-related variables, and demographic variables.

### Study population and data collection

Data from incident TMD cases aged ≥18 years were identified in the NPR during 1998 and 2016. The cases were identified using a TMD diagnosis (K07.6) and/or a surgical code used as a proxy for TMD. The following codes were used to identify the cases:

ICD-10 code:

- Temporomandibular joint disorders (K07.6)

Treatment codes:

- TMJ disc surgical procedure (EGB10)
- TMJ arthroscopy (EGA00)
- TMJ condylectomy (EGB00)
- TMJ prosthesis surgical procedure (EGC30)
- Other surgical reconstruction of TMJ (EGC99)
- TMJ synovectomy (EGB20)
- TMJ biopsy (EGA20)
- Injection of diagnostic or therapeutic substance in the TMJ (TEG10)
- TMJ condylotomy (EDC00)
- Open reposition of TMJ luxation (EGC00)
- TMJ plastic surgery (EGC10)
- TMJ plastic surgery with bone graft or other type of transplant (EGC20)

The cases were incident cases included on their first time of TMD diagnosis or surgical treatment, and later stratified into three groups: TMD diagnosis with no followed surgical treatment/non-surgical (NS); TMD-diagnosis with one surgical intervention (ST1); and TMD diagnosis with two or more surgical interventions (ST2). If a case received a diagnosis of K07.6 but had no subsequent surgical treatment, the case was assumed to be a patient with myofascial pain and/or dysfunction.

The controls were collected from the total population via the TPR and were matched for exact age, sex, living area, and being alive at the time of inclusion. Ten controls per case were included on the matched index date for each case.

Links between the registries were possible through the unique ten-digit Personal Identification Number, which is given to all Swedish citizens at birth, to immigrants upon becoming a citizen, receiving permanent residency status or other status that requires the recipient to pay income tax [17]. The exposures for cases and controls were collected through the NPR. Anonymized data were delivered by the NBHW, with individual serial numbers to enable cross-linking between registry data.

### Variables

#### Outcome

The outcome was incident TMD per the ICD code or surgical code definition during 1998 to 2016, in accordance with earlier description of the study population. Outcome was stratified into NS, ST1 and ST2 as described earlier.

#### Exposure

Exposure was defined as receiving one or several diagnoses corresponding to diagnostic codes within the 13^th^ chapter of ICD-10 (diseases of the musculoskeletal system and connective tissue/MSD). Diagnoses from the 7^th^, 8^th^, and 9^th^ version of ICD were translated using a codebook provided by the NBHW. For subjects who had received the same diagnosis multiple times, the exposure was only counted once–i.e., the first time it was given. Exposure variables were collected from 1964 until the date of receiving the outcome variable (cases) or inclusion in the study (controls). MSD diagnoses were classified into six diagnostic categories (1–6), and four of these were further subclassified into diagnostic groups (a–d), according to the ICD-10 classification system:

1. Arthropathies (M00–M25)
  1a) Infectious arthropathies (M00–M03)
  1b) Inflammatory polyarthropathies
  1c) Arthrosis
  1d) Other joint disorders (M20–M25)
2. Systemic connective tissue disorders (M30–M36)
3. Dorsopathies (M40–M54)
  3a) Deforming dorsopathies (M40–M43)
  3b) Spondylopathies (M45–M49)
  3c) Other dorsopathies (M50–M54)
4. Soft tissue disorders (M60–M79)
  4a) Disorders of the muscles (M60–M63)
  4b) Disorders of the synovium and tendon (M65–M68)
  4c) Other soft tissue disorders (M70–M79)
5. Osteopathies and chondropathies (M80–M94)
  5a) Disorders of bone density and structure (M80–M85)
  5b) Other osteopathies (M86–M90)
  5c) Chondropathies (M91–M94)
6. Other disorders of the musculoskeletal system and connective tissue (M95–M99)

#### Covariates

Level of education was classified according to number of years of education: primary and lower secondary school (0–9 years), upper secondary school (9–12 years), and post-secondary school (>12 years). Country of birth was classified as Sweden, Other Nordic countries, European countries, and non-European countries. To classify living area, Eurostat’s Degree of Urbanization (DEGURBA) was used (revised definition, 2016). Sweden consists of 290 municipalities, which were divided according to the DEGURBA classification: cities; towns or suburbs; and rural areas. The classification was conducted using Statistics Sweden’s publication on regional divisions in Sweden [18, 19].

As mental health comorbidity was considered a confounding factor, it was used as a binary adjustment variable. ICD codes corresponding to the 5^th^ chapter of ICD-10 mental health and behavioral disorders (MBD) were collected from the NPR and earlier codes from ICD-7, -8, and -9 were translated in the same manner as the main exposure variables. The diagnostic codes within the chapter (F00–F99) were dichotomized: 0 for no history of MBD and 1 for a history of MBD.

### Potential bias

Only exposures/diagnoses of MSD that had occurred before inclusion in the study were used to minimize risk for temporal bias. TMD patients that are diagnosed within the health care system but are not in need of surgical treatment comprise a small part of the entire TMD population, causing possible selection bias and affecting the generalizability, however the conclusions drawn from this study on this subset of TMD patients should not be affected by this. When using electronic medical records measurement bias should also be taken into account, due to various reasons such as erroneous typing or temporal changes of the journal systems [20]. There is however no reason to believe that these systematic biases would differ between the cases and controls, subsequently directing the bias towards the null.

### Statistical methods

Study sample size was not calculated a-priori due to the unpredictability of the size of the case-group. Data analysis was performed using STATA/SE 16.1, and odds ratios were calculated using conditional logistic regression [21]. Both outcome and exposure variables were considered dichotomous. The adjustment variables were included in the model as categorical dummy variables. Missing data for covariates were handled using Multiple Imputation by Chained Equations (MICE) [22], with 20 imputations and assuming missing at random. To impute on missing data with MICE, multinomial logistic regression was used for country of birth and DEGURBA, and ordinal logistic regression was used for educational level. The analyses were adjusted for level of education, country of birth, living area, and MBD comorbidity.

### Ethical declaration

The study was conducted in accordance with the Declaration of Helsinki and based on data Swedish national registries. The Swedish Ethical Review Authority approved the study (reference number 2018/401-31). The NBHW anonymized personal data before sending the data to the researchers. Informed consent is not required for registry-based research in accordance with the Swedish Ethical Review Authority.

## Results

### Characteristics of cases and controls

**Fig 1:**
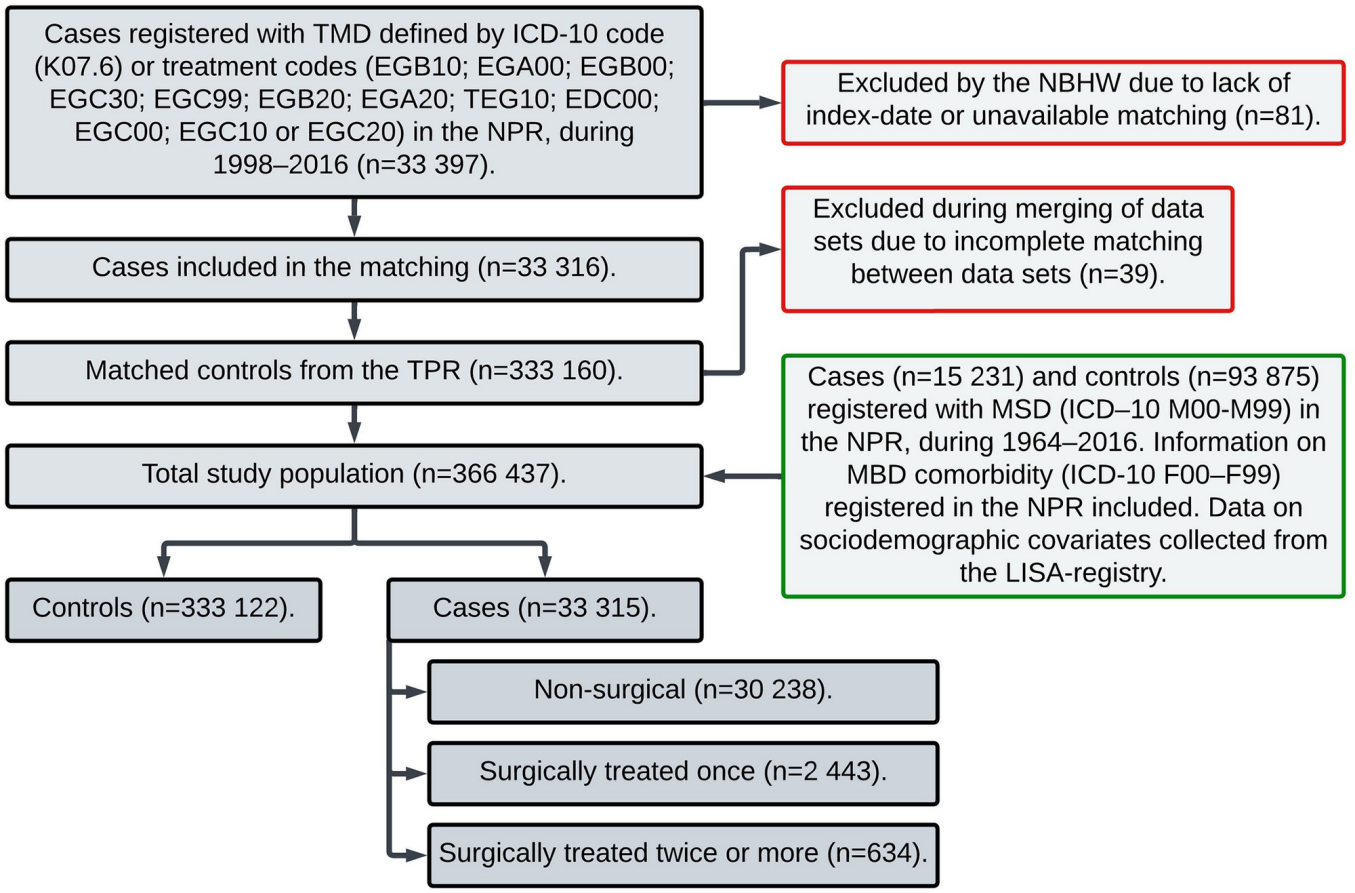
Recruitment flow-chart. Flow-chart of the recruitment process, Sweden, 1998–2016, 33 315 cases and 333 122 controls. The figure is a modified model of earlier published data from SWEREG-TMD [4]. ICD-10: International Classification of Diseases 10^th^ Revision. LISA: Longitudinal Integrated Database for Health Insurance and Labor Market Studies. NPR: National Patient Registry. TPR: Total Population Registry. MSD: Musculoskeletal and connective tissue diseases. MBD: Mental and behavioral disorders.

The recruitment process and exclusion of study subjects are described in Fig 1, which is a modified model of earlier presented data [4]. A total of 366 437 subjects were in the study population; of these, 33 315 cases and 333 122 controls were identified. The subgroups of the cases consisted of NS (n = 30 238), ST1 (n = 2443), and ST2 (n = 634). Most of the patients who had been surgically treated more than once had two surgical interventions (n = 450), and 184 patients had three or more surgical interventions, including three patients who had 9–12 surgical interventions.

The characteristics of the study population are presented in Table 1. Females constituted a majority in all subgroups of cases: NS 73%, ST1 77%, and ST2 84%. The mean age was 49 years for the entire case group as well as for the control group although markedly descending in the groups that had received surgical treatment once or multiple times. Educational level did not differ markedly between the cases and controls, and around half of the study population lived in cities. Most of the cases and controls were born in Sweden, with fewer subjects born outside of the Nordic countries and in non-European countries in the surgically treated groups. Table 1 also presents the MBD comorbidities for the cases and controls, with higher prevalence in the case groups for almost all MBD categories. The smallest difference in prevalence was found in mental retardation (F70–F79), and the highest prevalence of comorbidity for the entire study population were seen in mood affective disorders (F30–F39) and neurotic, stress-related, and somatoform disorders (F40–F48).

**Table 1:** Baseline and sociodemographic characteristics of the study population, as well as MBD comorbidity. Percentages are presented in parenthesis.

	Controls	Cases			
		All cases	NS^a^	ST1^b^	ST2^c^
	n = 333 122 (%)		n = 30 238 (%)	n = 2443 (%)	n = 634 (%)
**Sex**					
Female	242 868 (72.91)	24 289 (72.91)	21 887 (72.38)	1869 (76.50)	533 (84.07)
Male	90 254 (27.09)	9026 (27.09)	8351 (27.62)	574 (23.50)	101 (15.93)
**Educational level**					
Primary/lower secondary school	64 265 (19.29)	6467 (19.41)	6005 (19.86)	368 (15.06)	94 (14.83)
Upper secondary school	138 488 (41.57)	14 552 (43.68)	13 102 (43.33)	1149 (47.03)	301 (47.48)
Post-secondary school	125 833 (37.77)	11 989 (35.99)	10 837 (35.84)	914 (37.41)	238 (37.54)
Data unavailable	4536 (1.36)	307 (0.92)	294 (0.97)	12 (0.49)	1 (0.16)
**Country of birth**					
Sweden	269 829 (81.00)	25 885 (77.70)	23 222 (76.80)	2099 (85.92)	564 (88.96)
Other Nordic countries	11 772 (3.53)	1219 (3.66)	1099 (3.63)	97 (3.97)	23 (3.63)
Other European countries	22 960 (6.89)	2297 (6.89)	2192 (7.25)	87 (3.56)	18 (2.84)
Non-European countries	28 530 (8.56)	3913 (11.75)	3724 (12.32)	160 (6.55)	29 (4.57)
Data unavailable	31 (0.01)	1 (<0.00)	1 (<0.00)	0 (0)	0 (0)
**DEGURBA**					
Cities	150 065 (45.05)	14 631 (43.92)	13 092 (43.30)	1237 (50.63)	302 (47.63)
Towns and suburbs	89 917 (26.99)	9876 (29.64)	9106 (30.11)	597 (24.44)	173 (27.29)
Rural areas	90 570 (27.19)	8609 (25.84)	7861 (26.00)	591 (24.19)	157 (24.76)
Data unavailable	2570 (0.77)	199 (0.60)	179 (0.59)	18 (0.74)	2 (0.32)
**Age**					
Mean	49.15	49.15	49.96	41.62	39.31
IQR 25	34	34	35	26	25
Median	49	49	50	40	37
IQR 75	64	64	64	54	51
Range	18–104	18–104	18–104	18–97	18–89
**Marital status**					
Married	142 671 (42.83)	14 674 (44.05)	13 544 (44.79)	890 (36.43)	240 (37.85)
Not married	119 186 (35.78)	11 350 (34.07)	9899 (32.74)	1140 (46.66)	311 (49.05)
Divorced	46 442 (13.94)	5108 (15.33)	4720 (15.61)	320 (13.10)	68 (10.73)
Widow/widower	22 017 (6.61)	1952 (5.86)	1870 (6.18)	70 (2.87)	12 (1.89)
Other^d^	236 (0.07)	32 (0.10)	26 (0.09)	5 (0.20)	1 (0.16)
Data unavailable	2570 (0.77)	199 (0.60)	179 (0.59)	18 (0.74)	2 (0.32)
**Mental and behavioral disorders comorbidity**					
Any MBD^e^ comorbidity (F00-F99)	40 486 (12.15)	6201 (18.61)	5608 (18.55)	456 (18.67)	137 (21.61)
F00-F09 Organic, including symptomatic, mental disorders	6214 (1.87)	708 (2.13)	635 (2.10)	60 (2.46)	13 (2.05)
F10-F19 Mental and behavioral disorders due to psychoactive substance use	9919 (2.98)	1181 (3.54)	1034 (3.42)	117 (4.79)	30 (4.73)
F20-F29 Schizophrenia, schizotypal and delusional disorders	4044 (1.21)	436 (1.31)	385 (1.27)	41 (1.68)	10 (1.58)
F30-F39 Mood (affective) disorders	17 075 (5.13)	2635 (7.91)	2382 (7.88)	192 (7.86)	61 (9.62)
F40-F48 Neurotic, stress-related and somatoform disorders	22 271 (6.69)	4015 (12.05)	3656 (12.09)	273 (11.17)	86 (13.56)
F50-F59 Behavioral syndromes associated with physiological disturbances and physical factors	10 044 (3.02)	1523 (4.57)	1352 (4.47)	124 (5.08)	47 (7.41)
F60-F69 Disorders of adult personality and behavior	7684 (2.31)	1209 (3.63)	1076 (3.56)	99 (4.05)	34 (5.36)
F70-F79 Mental retardation	712 (0.21)	87 (0.26)	80 (0.26)	5 (0.20)	2 (0.32)
F80-F89 Disorders of psychological development	3603 (1.08)	496 (1.49)	426 (1.41)	48 (1.96)	22 (3.47)
F90-F98 Behavioral and emotional disorders with onset usually occurring in childhood and adolescence	8469 (2.54)	1343 (4.03)	1195 (3.95)	109 (4.46)	39 (6.15)
F99 Unspecified mental disorder	6884 (2.07)	981 (2.94)	871 (2.88)	80 (3.27)	30 (4.73)

NS^a^: Non-surgical.

ST1^b^: Surgically treated once.

ST2^c^: Surgically treated ≥2 times.

Other^d^: Registered partner, Divorced partner, Surviving partner.

MBD^e^. Mental and behavioral disorders.

**Fig 2:**
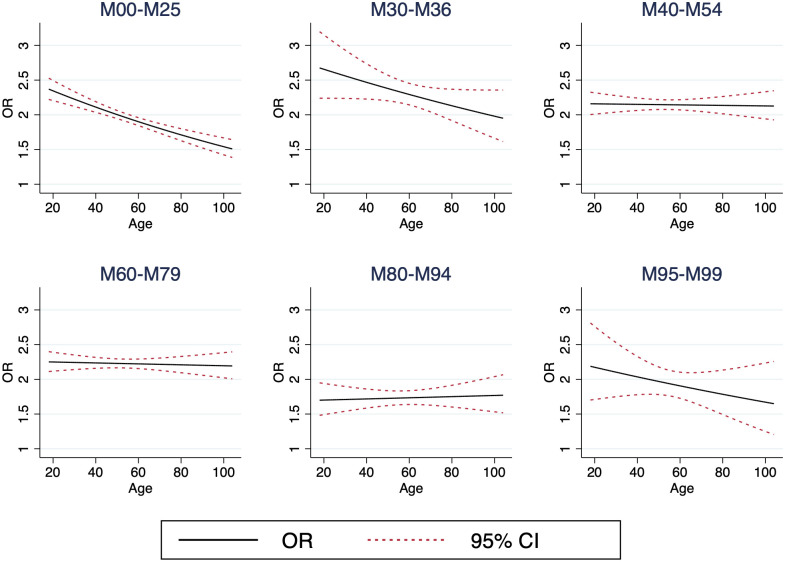
Interaction of age. Odds ratios in the six different diagnostic categories; Arthropathies (M00–M25), Systemic connective tissue disorders (M30–M36), Dorsopathies (M40–M54), Soft tissue disorders (M60–M79), Osteopathies and chondropathies (M80–M94), Other disorders of the musculoskeletal system and connective tissue (M95–M99) and the interaction with age. Odds ratios are calculated with conditional logistic regression and adjusted for educational level, country of birth, DEGURBA and MBD comorbidity.

An interaction analysis between the exposure of the six categories of MSD, the outcome TMD and age as a continuous variable was conducted. The results are presented in Fig 2. Odds ratios are calculated with conditional logistic regression and adjusted for all covariates.

### Main results

Table 2 present absolute numbers and frequencies of registered MSD among the controls and the three case subgroups. The most common diagnostic category among the controls was arthropathies (14.46%), which was followed by soft tissue disorders (13.14%). These diagnostic categories were also most common among the cases, with particularly higher numbers in the surgically treated groups, where 34.23% of the ST2 cases were suffering from arthropathies. A large difference was also seen in inflammatory polyarthropathies, with 2.29% among the controls compared to 16.56% in the ST2 case group. The most common diagnostic groups were other joint disorders (M20–M25) and other soft tissue disorders (M70–M79), with 21.61% and 23.03% respectively in the ST2 group. The least common diagnostic category was other disorders of the musculoskeletal system and connective tissue (M95–M99), with 0.82% in the controls and 1.59–2.01% in the case groups. Overall, the proportions of cases suffering from MSD increased in the subgroups, with generally the largest numbers in the ST2 subgroup.

**Table 2:** Crude numbers of individuals registered with arthropathies (M00–M25); systemic connective tissue disorders (M30–M36); dorsopathies (M40-–M54); soft tissue disorders (M60–M79); osteopathies and chondropathies (M80–M94) and other disorders of the musculoskeletal system and connective tissue (M95–M99). Percentages are presented in parenthesis.

	Controls	Cases		
		NS^a^	ST1^b^	ST2^c^
	n = 333 122 (%)	n = 30 238 (%)	n = 2443 (%)	n = 634 (%)
**1. M00-M25 Arthropathies**	**48 165 (14.46)**	**7090 (23.45)**	**702 (28.74)**	**217 (34.23)**
**1a) M00-M03 Infectious arthropathies**	**3089 (0.93)**	**477 (1.58)**	**78 (3.19)**	**21 (3.31)**
M00 Pyogenic Arthritis	2310 (0.69)	343 (1.13)	49 (2.01)	15 (2.37)
M01 Direct infections of joint in infectious and parasitic diseases classified elsewhere	151 (0.05)	19 (0.06)	5 (0.20)	1 (0.16)
M02 Reactive arthropathies	1988 (0.60)	306 (1.01)	53 (2.17)	14 (2.21)
M03 Post-infective and reactive arthropathies in diseases classified elsewhere	23 (0.01)	6 (0.02)	0 (0)	0 (0)
**1b) M05-M14 Inflammatory polyarthropathies**	**7626 (2.29)**	**1371 (4.53)**	**264 (10.81)**	**105 (16.56)**
M05 Seropositive rheumatoid arthritis	2567 (0.77)	499 (1.65)	91 (3.72)	38 (5.99)
M06 Other rheumatoid arthritis	2358 (0.71)	463 (1.53)	102 (4.18)	38 (5.99)
M07 Psoriatic and enteropathic arthropathies	64 (0.02)	16 (0.05)	6 (0.25)	1 (0.16)
M08 Juvenile arthritis	362 (0.11)	112 (0.37)	65 (2.66)	29 (4.57)
M09 Juvenile arthritis in diseases classified elsewhere	103 (0.03)	25 (0.08)	13 (0.53)	6 (0.95)
M10 Gout	623 (0.19)	83 (0.27)	7 (0.29)	1 (0.16)
M11 Other crystal arthropathies	612 (0.18)	102 (0.34)	17 (0.70)	1 (0.16)
M12 Other specific arthropathies	1997 (0.60)	324 (1.07)	62 (2.54)	21 (3.31)
M13 Other arthritis	3614 (1.08)	650 (2.15)	137 (5.61)	47 (7.41)
M14 Arthropathies in other diseases classified elsewhere	79 (0.02)	14 (0.05)	1 (0.04)	4 (0.63)
**1c) M15-M19 Arthrosis**	**20 675 (6.21)**	**2948 (9.75)**	**222 (9.09)**	**63 (9.94)**
M15 Polyarthrosis	2105 (0.63)	382 (1.26)	43 (1.76)	13 (2.05)
M16 Coxarthrosis	6663 (2.00)	792 (2.62)	49 (2.01)	13 (2.05)
M17 Gonarthrosis	9851 (2.96)	1430 (4.73)	72 (2.95)	21 (3.31)
M18 Arthrosis of first carpometacarpal joint	2209 (0.66)	383 (1.27)	26 (1.06)	12 (1.89)
M19 Other arthrosis	6125 (1.84)	1041 (3.44)	119 (4.87)	36 (5.68)
**1d) M20-M25 Other joint disorders**	**29 890 (8.97)**	**4632 (15.32)**	**439 (17.97)**	**137 (21.61)**
M20 Acquired deformities of fingers and toes	7242 (2.17)	1090 (3.60)	74 (3.03)	16 (2.52)
M21 Other acquired deformities of limbs	2594 (0.78)	367 (1.21)	40 (1.64)	14 (2.21)
M22 Disorders of patella	4593 (1.38)	679 (2.25)	80 (3.27)	31 (4.89)
M23 Internal derangement of knee	10 668 (3.20)	1414 (4.68)	123 (5.03)	40 (6.31)
M24 Other specific joint derangements	5179 (1.55)	769 (2.54)	122 (4.99)	51 (8.04)
M25 Other joint disorders, not elsewhere classified	11 916 (3.58)	2195 (7.26)	221 (9.05)	82 (12.93)
**2. M30-M36 Systemic connective tissue disorders**	**4852 (1.46)**	**963 (3.18)**	**96 (3.93)**	**32 (5.05)**
M30 Polyarteritis nodosa and related conditions	47 (0.01)	10 (0.03)	0 (0)	0 (0)
M31 Other necrotizing vasculopathies	679 (0.20)	194 (0.64)	12 (0.49)	5 (0.79)
M32 Systemic lupus erythematosus	388 (0.12)	64 (0.21)	8 (0.33)	3 (0.47)
M33 Dermatopolymyositis	123 (0.04)	19 (0.06)	3 (0.12)	0 (0)
M34 Systemic sclerosis	151 (0.05)	31 (0.10)	3 (0.12)	0 (0)
M35 Other systemic involvement of connective tissue	2970 (0.89)	618 (2.04)	47 (1.92)	24 (3.79)
M36 Systemic disorders of connective tissue in diseases classified elsewhere	1539 (0.46)	212 (0.70)	46 (1.88)	10 (1.58)
**3. M40-M54 Dorsopathies**	**28 592 (8.58)**	**5173 (17.11)**	**414 (16.95)**	**129 (20.35)**
**3a) M40-M43 Deforming dorsopathies**	**4590 (1.38)**	**712 (2.35)**	**91 (3.72)**	**28 (4.42)**
M40 Kyphosis and lordosis	960 (0.29)	116 (0.38)	22 (0.90)	8 (1.26)
M41 Scoliosis	1309 (0.39)	142 (0.47)	19 (0.78)	6 (0.95)
M42 Spinal osteochondrosis	48 (0.01)	7 (0.02)	2 (0.08)	2 (0.32)
M43 Other deforming dorsopathies	3484 (1.05)	586 (1.94)	69 (2.82)	18 (2.84)
**3b) M45-M49 Spondylopathies**	**7664 (2.30)**	**1377 (4.55)**	**121 (4.95)**	**38 (5.99)**
M45 Ankylosing spondylitis	1996 (0.60)	352 (1.16)	51 (2.09)	14 (2.21)
M46 Other inflammatory spondylopathies	3005 (0.90)	535 (1.77)	78 (3.19)	22 (3.47)
M47 Spondylosis	3052 (0.92)	586 (1.94)	49 (2.01)	15 (2.37)
M48 Other spondylopathies	5074 (1.52)	828 (2.74)	65 (2.66)	20 (3.15)
M49 Spondylopathies in diseases classified elsewhere	63 (0.02)	11 (0.04)	0 (0)	0 (0)
**3c) M50-M54 Other dorsopathies**	**23 374 (7.02)**	**4431 (14.65)**	**334 (13.67)**	**104 (16.40)**
M50 Cervical disc disorders	2226 (0.67)	412 (1.36)	41 (1.68)	11 (1.74)
M51 Thoracic, thoracolumbar, and lumbosacral intervertebral disc disorders	4983 (1.50)	791 (2.62)	66 (2.70)	21 (3.31)
M53 Other and unspecified dorsopathies, not elsewhere classified	3835 (1.15)	769 (2.54)	71 (2.91)	22 (3.47)
M54 Dorsalgia	19 320 (5.80)	3783 (12.51)	291 (11.91)	85 (13.41)
**4. M60-M79 Soft tissue disorders**	**43 775 (13.14)**	**7605 (25.15)**	**511 (20.92)**	**164 (25.87)**
**4a) M60-M63 Disorders of the muscles**	**3202 (0.96)**	**560 (1.85)**	**38 (1.56)**	**20 (3.15)**
M60 Myositis	1674 (0.50)	301 (1.00)	24 (0.98)	14 (2.21)
M61 Calcification and ossification of muscle	249 (0.07)	34 (0.11)	2 (0.08)	1 (0.16)
M62 Other disorders of muscle	2631 (0.79)	425 (1.41)	32 (1.31)	16 (2.52)
M63 Disorders of muscle in diseases classified elsewhere	3 (0.00)	0 (0)	0 (0)	0 (0)
**4b) M65-M68 Disorders of the synovium and tendon**	**10 268 (3.08)**	**1640 (5.42)**	**121 (4.95)**	**50 (7.89)**
M65 Synovitis and tenosynovitis	6477 (1.94)	1048 (3.47)	93 (3.81)	38 (5.99)
M66 Spontaneous rupture of synovium and tendon	1793 (0.54)	288 (0.95)	18 (0.74)	11 (1.74)
M67 Other disorders of synovium and tendon	4208 (1.26)	677 (2.24)	40 (1.64)	22 (3.47)
M68 Disorders of synovium and tendon in diseases classified elsewhere	870 (0.26)	139 (0.46)	12 (0.49)	7 (1.10)
**4c) M70-M79 Other soft tissue disorders**	**36 737 (11.03)**	**6660 (22.03)**	**446 (18.26)**	**146 (23.03)**
M70 Soft tissue disorders related to use, overuse and pressure	4190 (1.26)	744 (2.46)	56 (2.29)	23 (3.63)
M71 Other bursopathies	2525 (0.76)	390 (1.29)	37 (1.51)	19 (3.00)
M72 Fibroblastic disorders	1836 (0.55)	316 (1.05)	18 (0.74)	6 (0.95)
M73 Soft tissue disorders in diseases classified elsewhere	837 (0.25)	145 (0.48)	12 (0.49)	8 (1.26)
M75 Shoulder lesions	7397 (2.22)	1337 (4.42)	105 (4.30)	32 (5.05)
M76 Enthesopathies, lower limb, excluding foot	3403 (1.02)	573 (1.89)	43 (1.76)	20 (3.15)
M77 Other enthesopathies	5188 (1.56)	934 (3.09)	78 (3.19)	37 (5.84)
M79 Other and unspecified soft tissue disorders, not elsewhere classified	24917 (7.48)	4894 (16.18)	316 (12.93)	104 (16.40)
**5. M80-M94 Osteopathies and chondropathies**	**9104 (2.73)**	**1406 (4.65)**	**107 (4.38)**	**40 (6.31)**
**5a) M80-M85 Disorders of bone density and structure**	**5314 (1.60)**	**824 (2.73)**	**56 (2.29)**	**21 (3.31)**
M80 Osteoporosis with pathological fracture	1121 (0.34)	172 (0.57)	5 (0.20)	5 (0.79)
M81 Osteoporosis without pathological fracture	2444 (0.73)	385 (1.27)	24 (0.98)	12 (1.89)
M82 Osteoporosis in diseases classified elsewhere	691 (0.21)	90 (0.30)	14 (0.57)	5 (0.79)
M83 Adult osteomalacia	103 (0.03)	23 (0.08)	2 (0.08)	1 (0.16)
M84 Disorders of continuity of bone	2039 (0.61)	281 (0.93)	35 (1.43)	9 (1.42)
M85 Other disorders of bone density and structure	1812 (0.54)	274 (0.91)	26 (1.06)	9 (1.42)
**5b) M86-M90 Other osteopathies**	**3268 (0.98)**	**482 (1.59)**	**53 (2.17)**	**21 (3.31)**
M86 Osteomyelitis	1564 (0.47)	197 (0.65)	30 (1.23)	9 (1.42)
M87 Osteonecrosis	1287 (0.39)	117 (0.59)	21 (0.86)	6 (0.95)
M88 Osteitis deformans (Paget’s disease of bone)	13 (0.00)	5 (0.02)	0 (0)	0 (0)
M89 Other disorders of bone	2167 (0.65)	332 (1.10)	38 (1.56)	19 (3.00)
M90 Osteopathies in diseases classified elsewhere	1022 (0.31)	139 (0.46)	19 (0.78)	6 (0.95)
**5c) M91-M94 Chondropathies**	**2893 (0.87)**	**437 (1.45)**	**41 (1.68)**	**16 (2.52)**
M91 Juvenile osteochondrosis of hip and pelvis	1117 (0.34)	135 (0.45)	20 (0.82)	9 (1.42)
M92 Other juvenile osteochondrosis	776 (0.23)	94 (0.31)	10 (0.41)	6 (0.95)
M93 Other osteochondropathies	625 (0.19)	60 (0.20)	2 (0.08)	4 (0.63)
M94 Other disorders of cartilage	1678 (0.50)	310 (1.03)	29 (1.19)	9 (1.42)
**6. M95-M99 Other disorders of the musculoskeletal system and connective tissue**	**2736 (0.82)**	**482 (1.59)**	**49 (2.01)**	**12 (1.89)**
M95 Other acquired deformities of musculoskeletal system and connective tissue	1001 (0.30)	168 (0.56)	14 (0.57)	4 (0.63)
M96 Postprocedural musculoskeletal disorders, not elsewhere classified	1569 (0.47)	225 (0.74)	32 (1.31)	8 (1.26)
M99 Biomechanical lesions, not elsewhere classified	470 (0.14)	123 (0.41)	6 (0.25)	1 (0.16)

NS^a^: Non-surgical.

ST1^b^: Surgically treated once.

ST2^c^: Surgically treated ≥2 times.

Table 3 presents the odds ratios of developing TMD following MSD diagnosis, calculated with conditional logistic regression. For the entire case group, there was an overall doubled odds for all the diagnostic categories. For all diagnostic categories and groups, the association between MSD and TMD was increasingly stronger in the surgically treated subgroups, in particular the subgroup with multiple surgical intervention. The diagnostic category with the strongest association was arthropathies (M00-M25) with an OR of 5.6 (CI 4.6–6.9) for the ST2 subgroup. For inflammatory polyarthropathies, a diagnostic group within arthropathies, the OR was 11.7 (CI 8.6–15.9). All results for the diagnostic categories and groups were significant, but no significant association between MSD and TMD was found for some specific diagnostic codes, with lack of significance either for the entire case group or for specific case subgroups.

**Table 3:** Results from conditional logistic regression adjusted for educational level, DEGURBA, and year of diagnosis. Odds ratios presented for arthropathies (M00–M25); systemic connective tissue disorders (M30–M36); dorsopathies (M40–M54); soft tissue disorders (M60–M79); osteopathies and chondropathies (M80–M94) and other disorders of the musculoskeletal system and connective tissue (M95–M99).

	All cases		NS^a^		ST1^b^		ST2^c^	
	OR	(95% CI)	OR	(95% CI)	OR	(95% CI)	OR	(95% CI)
**1. M00-M25 Arthropathies (M00-M25)**	**2.0**	**(1.9 2.0)**	**1.8**	**(1.8 1.9)**	**4.0**	**(3.6 4.4)**	**5.6**	**(4.6 6.9)**
**1a) M00-M03 Infectious arthropathies**	**1.9**	**(1.7 2.0)**	**1.7**	**(1.5 1.8)**	**4.1**	**(3.1 5.4)**	**3.8**	**(2.2 6.5)**
M00 Pyogenic Arthritis	1.8	(1.6 1.9)	1.6	(1.4 1.8)	3.3	(2.4 4.7)	3.3	(1.8 6.0)
M01 Direct infections of joint in infectious and parasitic diseases classified elsewhere	1.6	(1.0 2.4)	1.3^d^	(0.8 2.2)	3.7	(1.3 10.5)	2.2^d^	(0.2 26.4)
M02 Reactive arthropathies	1.9	(1.7 2.1)	1.7	(1.5 1.9)	4.1	(2.9 5.8)	3.8	(2.0 7.4)
M03 Post-infective and reactive arthropathies in diseases classified elsewhere	2.1^d^	(0.9 5.2)	2.8	(1.1 7.2)	^e^	.	^e^	.
**1b) M05-M14 Inflammatory polyarthropathies**	**2.4**	**(2.2 2.5)**	**2.0**	**(1.9 2.1)**	**6.9**	**(5.8 8.1)**	**11.7**	**(8.6 15.9)**
M05 Seropositive rheumatoid arthritis	2.5	(2.3 2.7)	2.1	(1.9 2.3)	7.9	(6.0 10.5)	10.4	(6.4 16.9)
M06 Other rheumatoid arthritis	2.6	(2.4 2.9)	2.1	(1.9 2.4)	9.7	(7.3 12.8)	10.0	(6.2 16.0)
M07 Psoriatic and enteropathic arthropathies	3.5	(2.2 5.7)	2.6	(1.5 4.5)	27.2	(5.4 137.0)	^e^	.
M08 Juvenile arthritis	5.9	(5.0 7.0)	3.7	(2.0 4.6)	18.9	(12.2 29.1)	32.7	(15.2 70.1)
M09 Juvenile arthritis in diseases classified elsewhere	4.4	(3.1 6.2)	2.9	(1.8 4.5)	10.7	(4.8 24.1)	47.5	(5.7 398.7)
M10 Gout	1.4	(1.1 1.7)	1.3	(1.1 1.7)	3.3	(1.4 8.0)	1.6^d^	(0.2 14.1)
M11 Other crystal arthropathies	2.0	(1.6 2.4)	1.8	(1.5 2.2)	4.8	(2.7 8.6)	2.4^d^	(0.3 20.8)
M12 Other specific arthropathies	2.0	(1.8 2.3)	1.8	(1.6 2.0)	4.5	(3.3 6.2)	4.9	(2.8 8.5)
M13 Other arthritis	2.3	(2.2 2.5)	2.0	(1.8 2.2)	6.0	(4.8 7.4)	7.9	(5.2 12.0)
M14 Arthropathies in other diseases classified elsewhere	2.6	(1.6 4.3)	2.0	(1.1 3.6)	3.7^c^	(0.4 35.4)	18.5	(3.3 102.6)
**1c) M15-M19 Arthrosis**	**1.7**	**(1.6 1.8)**	**1.6**	**(1.6 1.7)**	**3.3**	**(2.8 3.9)**	**4.2**	**(3.0 5.9)**
M15 Polyarthrosis	2.1	(1.9 2.3)	2.0	(1.8 2.2)	4.0	(2.8 5.7)	4.2	(2.1 8.4)
M16 Coxarthrosis	1.3	(1.2 1.4)	1.3	(1.2 1.4)	2.1	(1.5 2.9)	1.9	(1.0 3.6)
M17 Gonarthrosis	1.6	(1.5 1.7)	1.6	(1.5 1.7)	2.1	(1.6 2.7)	3.4	(2.0 5.8)
M18 Arthrosis of first carpometacarpal joint	1.9	(1.7 2.1)	1.8	(1.6 2.0)	3.0	(1.9 4.7)	4.5	(2.2 9.2)
M19 Other arthrosis	2.0	(1.9 2.1)	1.8	(1.7 2.0)	5.2	(4.1 6.5)	6.6	(4.2 10.5)
**1d) M20-M25 Other joint disorders**	**1.9**	**(1.8 2.0)**	**1.8**	**(1.8 1.9)**	**3.1**	**(2.7 3.5)**	**3.9**	**(3.1 4.8)**
M20 Acquired deformities of fingers and toes	1.7	(1.6 1.8)	1.6	(1.5 1.7)	2.2	(1.7 2.8)	1.5^c^	(0.9 2.6)
M21 Other acquired deformities of limbs	1.6	(1.4 1.8)	1.5	(1.4 1.7)	2.4	(1.7 3.5)	4.3	(2.2 8.2)
M22 Disorders of patella	1.7	(1.6 1.9)	1.6	(1.5 1.8)	2.6	(2.0 3.4)	3.3	(2.1 5.0)
M23 Internal derangement of knee	1.5	(1.5 1.6)	1.5	(1.4 1.6)	2.1	(1.7 2.6)	2.5	(1.8 3.7)
M24 Other specific joint derangements	1.8	(1.7 2.0)	1.6	(1.5 1.8)	3.8	(3.1 4.7)	8.1	(5.4 12.0)
M25 Other joint disorders, not elsewhere classified	2.2	(2.1 2.3)	2.1	(2.0 2.2)	3.4	(2.9 4.0)	5.0	(3.8 6.8)
**2. M30-M36 Systemic connective tissue disorders**	**2.3**	**(2.1 2.4)**	**2.2**	**(2.0 2.3)**	**3.4**	**(2.7 4.3)**	**4.8**	**(3.1 7.5)**
M30 Polyarteritis nodosa and related conditions	2.1	(1.1 4.3)	2.4	(1.2 4.8)	^e^	.	^e^	.
M31 Other necrotizing vasculopathies	3.1	(2.6 3.6)	2.9	(2.5 3.5)	5.5	(2.6 11.4)	34.2	(6.3 186.4)
M32 Systemic lupus erythematosus	1.9	(1.5 2.4)	1.8	(1.4 2.3)	2.6	(1.2 5.6)	6.6	(1.4 30.3)
M33 Dermatopolymyositis	1.8	(1.1 2.8)	1.6^d^	(1.0 2.6)	6.5	(1.4 29.2)	^e^	.
M34 Systemic sclerosis	2.2	(1.5 3.2)	2.2	(1.5 3.2)	4.1	(1.0 16.4)	^e^	.
M35 Other systemic involvement of connective tissue	2.3	(2.1 2.5)	2.2	(2.0 2.4)	2.8	(2.0 4.0)	5.4	(3.3 9.1)
M36 Systemic disorders of connective tissue in diseases classified elsewhere	1.8	(1.5 2.0)	1.6	(1.3 1.8)	3.8	(2.7 5.4)	2.7	(1.3 5.8)
**3. M40-M54 Dorsopathies**	**2.2**	**(2.1 2.2)**	**2.1**	**(2.0 2.2)**	**2.9**	**(2.6 3.3)**	**4.0**	**(3.1 5.0)**
**3a) M40-M43 Deforming dorsopathies**	**1.8**	**(1.7 1.9)**	**1.7**	**(1.6 1.8)**	**2.4**	**(1.9 3.1)**	**2.8**	**(1.8 4.4)**
M40 Kyphosis and lordosis	1.5	(1.3 1.8)	1.4	(1.1 1.7)	2.5	(1.5 4.0)	3.2	(1.4 7.3)
M41 Scoliosis	1.3	(1.1 1.5)	1.3	(1.1 1.5)	1.3^d^	(0.8 2.1)	1.7^d^	(0.7 4.3)
M42 Spinal osteochondrosis	2.2	(1.1 4.2)	1.7^d^	(0.8 3.8)	2.9^d^	(0.6 14.2)	11.2^d^	(1.0 132.0)
M43 Other deforming dorsopathies	1.9	(1.7 2.1)	1.8	(1.6 2.0)	2.8	(2.1 3.7)	2.6	(1.5 4.6)
**3b) M45-M49 Spondylopathies**	**2.0**	**(1.9 2.1)**	**1.9**	**(1.8 2.0)**	**3.2**	**(2.6 4.0)**	**5.1**	**(3.4 7.7)**
M45 Ankylosing spondylitis	2.1	(1.9 2.3)	1.9	(1.7 2.1)	4.0	(2.9 5.6)	3.6	(1.9 6.9)
M46 Other inflammatory spondylopathies	2.1	(1.9 2.3)	2.0	(1.7 2.1)	4.3	(3.2 5.6)	5.2	(3.1 8.8)
M47 Spondylosis	2.1	(1.9 2.3)	2.0	(1.9 2.2)	2.7	(1.9 3.7)	3.1	(1.7 5.7)
M48 Other spondylopathies	1.8	(1.7 1.9)	1.7	(1.6 1.9)	2.7	(2.0 3.5)	3.8	(2.2 6.6)
M49 Spondylopathies in diseases classified elsewhere	1.8^d^	(0.9 3.4)	2.0	(1.0 3.7)	^e^	.	^e^	.
**3c) M50-M54 Other dorsopathies**	**2.2**	**(2.1 2.3)**	**2.1**	**(2.1 2.2)**	**2.9**	**(2.5 3.3)**	**3.7**	**(2.9 4.8)**
M50 Cervical disc disorders	2.0	(1.8 2.2)	1.9	(1.7 2.1)	3.2	(2.2 4.6)	3.2	(1.6 6.4)
M51 Thoracic, thoracolumbar, and lumbosacral intervertebral disc disorders	1.7	(1.6 1.9)	1.7	(1.6 1.8)	2.5	(1.9 3.3)	2.5	(1.5 4.0)
M53 Other and unspecified dorsopathies, not elsewhere classified	2.2	(2.0 2.3)	2.1	(1.9 2.3)	2.9	(2.2 3.8)	3.7	(2.2 6.2)
M54 Dorsalgia	2.2	(2.1 2.3)	2.2	(2.1 2.2)	3.0	(2.6 3.4)	3.5	(2.6 4.6)
**4. M60-M79 Soft tissue disorders**	**2.2**	**(2.2 2.3)**	**2.2**	**(2.1 2.3)**	**2.7**	**(2.4 3.0)**	**3.7**	**(3.0 4.6)**
**4a) M60-M63 Disorders of the muscles**	**1.9**	**(1.7 2.1)**	**1.9**	**(1.7 2.0)**	**1.9**	**(1.3 2.7)**	**3.6**	**(2.1 6.2)**
M60 Myositis	2.0	(1.7 2.2)	1.9	(1.7 2.2)	2.1	(1.3 3.3)	4.8	(2.5 9.2)
M61 Calcification and ossification of muscle	1.5	(1.0 2.1)	1.5	(1.0 2.1)	1.1^d^	(0.2 4.8)	5.1^d^	(0.5 56.9)
M62 Other disorders of muscle	1.8	(1.6 2.0)	1.7	(1.6 1.9)	1.9	(1.3 2.9)	3.4	(1.9 6.2)
M63 Disorders of muscle in diseases classified elsewhere	^e^	.	^e^	.	^e^	.	^e^	.
**4b) M65-M68 Disorders of the synovium and tendon**	**1.8**	**(1.7 1.9)**	**1.8**	**(1.7 1.8)**	**2.2**	**(1.8 2.7)**	**3.5**	**(2.5 4.9)**
M65 Synovitis and tenosynovitis	1.8	(1.7 2.0)	1.8	(1.6 1.9)	2.7	(2.1 3.4)	4.3	(2.9 6.5)
M66 Spontaneous rupture of synovium and tendon	1.8	(1.6 2.0)	1.7	(1.5 2.0)	1.8	(1.1 3.1)	4.9	(2.3 10.5)
M67 Other disorders of synovium and tendon	1.8	(1.6 1.9)	1.7	(1.6 1.9)	1.6	(1.2 2.3)	3.3	(2.0 5.4)
M68 Disorders of synovium and tendon in diseases classified elsewhere	1.8	(1.5 2.1)	1.7	(1.4 2.1)	2.3	(1.2 4.3)	4.2	(1.6 10.9)
**4c) M70-M79 Other soft tissue disorders**	**2.3**	**(2.2 2.3)**	**2.2**	**(2.2 2.3)**	**2.8**	**(2.4 3.1)**	**3.9**	**(3.1 4.9)**
M70 Soft tissue disorders related to use, overuse and pressure	2.0	(1.8 2.1)	1.9	(1.8 2.1)	2.4	(1.7 3.2)	3.6	(2.2 5.9)
M71 Other bursopathies	1.8	(1.6 1.9)	1.7	(1.5 1.9)	2.3	(1.6 3.4)	4.7	(2.6 8.3)
M72 Fibroblastic disorders	1.8	(1.6 2.1)	1.8	(1.6 2.1)	1.9	(1.1 3.2)	4.5	(1.7 12.3)
M73 Soft tissue disorders in diseases classified elsewhere	1.9	(1.6 2.3)	1.8	(1.5 2.2)	2.3	(1.2 4.4)	6.1	(2.3 15.7)
M75 Shoulder lesions	2.0	(1.9 2.2)	2.0	(1.8 2.1)	3.4	(2.7 4.2)	3.8	(2.4 5.8)
M76 Enthesopathies, lower limb, excluding foot	1.9	(1.7 2.0)	1.8	(1.7 2.0)	2.2	(1.6 3.1)	4.0	(2.3 6.9)
M77 Other enthesopathies	2.0	(1.9 2.2)	1.9	(1.8 2.1)	2.5	(2.0 3.3)	4.8	(3.2 7.3)
M79 Other and unspecified soft tissue disorders, not elsewhere classified	2.3	(2.2 2.4)	2.3	(2.2 2.4)	2.6	(2.3 3.0)	3.4	(2.7 4.4)
**5. M80-M94 Osteopathies and chondropathies**	**1.7**	**(1.6 1.8)**	**1.7**	**(1.6 1.8)**	**2.2**	**(1.7 2.7)**	**3.6**	**(2.5 5.4)**
**5a) M80-M85 Disorders of bone density and structure**	**1.7**	**(1.6 1.8)**	**1.7**	**(1.5 1.8)**	**2.2**	**(1.7 3.0)**	**3.7**	**(2.1 6.3)**
M80 Osteoporosis with pathological fracture	1.6	(1.4 1.9)	1.6	(1.4 1.9)	1.3^d^	(0.5 3.2)	9.5	(2.4 37.3)
M81 Osteoporosis without pathological fracture	1.7	(1.6 1.9)	1.7	(1.5 1.9)	2.0	(1.3 3.2)	3.3	(1.6 6.6)
M82 Osteoporosis in diseases classified elsewhere	1.6	(1.3 1.9)	1.4	(1.2 1.8)	2.6	(1.4 4.8)	2.6^d^	(1.0 7.2)
M83 Adult osteomalacia	2.4	(1.6 3.7)	2.3	(1.4 3.6)	3.2^a^	(0.6 16.1)	9.5^d^	(0.6 162.6)
M84 Disorders of continuity of bone	1.6	(1.4 1.7)	1.5	(1.3 1.7)	2.8	(1.9 4.1)	3.1	(1.4 6.7)
M85 Other disorders of bone density and structure	1.7	(1.5 1.9)	1.6	(1.4 1.9)	2.4	[23]	3.0	(1.4 6.4)
**5b) M86-M90 Other osteopathies**	**1.7**	**(1.5 1.8)**	**1.6**	**(1.4 1.7)**	**2.6**	**(1.9 3.5)**	**4.0**	**(2.3 6.7)**
M86 Osteomyelitis	1.5	(1.3 1.7)	1.4	(1.2 1.6)	2.8	(1.9 4.3)	2.9	(1.3 6.1)
M87 Osteonecrosis	1.6	(1.4 1.8)	1.5	(1.3 1.8)	2.4	(1.5 3.9)	2.2^d^	(0.9 5.5)
M88 Osteitis deformans (Paget’s disease of bone)	3.7	(1.3 10.6)	3.7	(1.3 10.5)	^e^	.	^e^	.
M89 Other disorders of bone	1.8	(1.6 2.0)	1.7	(1.5 1.9)	2.5	(1.8 3.7)	5.2	(2.9 9.3)
M90 Osteopathies in diseases classified elsewhere	1.6	(1.4 1.9)	1.5	(1.3 1.8)	2.5	(1.5 4.1)	2.6	(1.0 6.5)
**5c) M91-M94 Chondropathies**	**1.7**	**(1.5 1.9)**	**1.6**	**(1.5 1.8)**	**1.9**	**(1.4 2.7)**	**3.3**	**(1.8 5.9)**
M91 Juvenile osteochondrosis of hip and pelvis	1.5	(1.2 1.7)	1.4	(1.1 1.6)	2.1	(1.3 3.4)	3.6	(1.6 8.0)
M92 Other juvenile osteochondrosis	1.4	(1.2 1.8)	1.4	(1.1 1.7)	1.6^d^	(0.8 3.2)	4.8	(1.8 12.9)
M93 Other osteochondropathies	1.1^d^	(0.8 1.4)	1.1^d^	(0.8 1.4)	0.4^d^	(0.1 1.5)	7.9	(2.0 30.2)
M94 Other disorders of cartilage	2.0	(1.8 2.2)	1.9	(1.7 2.2)	2.7	(1.8 4.0)	2.7	(1.2 5.8)
**6. M95-M99 Other disorders of the musculoskeletal system and connective tissue**	**1.9**	**(1.8 2.1)**	**1.9**	**(1.7 2.1)**	**2.7**	**(1.9 3.7)**	**2.9**	**(1.5 5.7)**
M95 Other acquired deformities of musculoskeletal system and connective tissue	1.8	(1.5 2.1)	1.8	(1.5 2.1)	1.6^d^	(0.9 2.8)	3.3	(1.0 10.6)
M96 Postprocedural musculoskeletal disorders, not elsewhere classified	1.7	(1.5 1.9)	1.6	(1.4 1.8)	3.1	(2.0 4.6)	2.7	(1.2 6.1)
M99 Biomechanical lesions, not elsewhere classified	2.6	(2.1 3.2)	2.6	(2.1 3.2)	2.4^d^	(0.9 6.2)	2.2^d^	(0.2 20.0)

NS^a^: Non-surgical.

ST1^b^: Surgically treated once.

ST2^c^: Surgically treated ≥2 times.

^d^: P>0.05.

^e^: Omitted due to too few observations.

## Discussion

Our study showed a significant and substantial association between MSD and the development of TMD. The odds of developing TMD following any MSD diagnosis were approximately doubled for all diagnostic categories of MSD, with much stronger associations in the surgically treated subgroups, specifically cases with multiple surgical interventions. This highlights that the group with multiple surgical interventions also represent patients with the most severe type of TMD, joint involvement and disease burden. Although some diagnoses are heterogeneous and the causal relationship is not clear, there is a stable statistically significant increase in potential risk that cannot be ignored. It is obvious that there is a ladder effect throughout the results, where surgically treated patients have a severely increased disease burden. The effects of these results are multifaceted. As the diagnoses are common in the general population in relation to the association with TMD, improved early interventions should lower the number of patients requiring surgical treatment, reducing unnecessary medical procedures and risk of post-surgical complications. Considering the formidable impact on societal costs that TMD patients with multiple surgical interventions bestow on the society, a reduction in the number of these patients should therefore have a substantial effect on reducing societal costs associated with the treatment [4].

Indicators of general poor health have been found to be important in predicting TMD, but population-based studies using registry data have not been conducted earlier [5, 24]. The aim of the scientific studies based on SWEREG-TMD is to contribute with unique, validated, and epidemiological data to shed further light on potential risk factors associated with the development of TMD, to aid in identifying areas for prevention and evidence-based treatment related to this highly complex and vulnerable patient group. Our results emphasize the importance of the interaction between odontological and medical specialists. Many TMD patients are referred to oral and maxillofacial surgeons from the dental care system, and it is plausible that many of these patients who have been conservatively treated have an unknown or undiagnosed underlying systemic disease that contributes to the failure of less invasive treatment methods and therefore referred to surgery. In these cases, management and treatment of the underlying disease should be initiated before considering surgery to avoid unnecessary interventions or improve the outcomes of surgery. Therefore, dentists ought to be attentive to anamnestic information that might imply underlying comorbidity and ensure that these patients are properly examined by a medical professional. In addition, medical doctors should be aware of the potential risks of developing TMD within this patient group to detect early symptoms of TMD. Such early intervention with referral to a dentist or a specialist in orofacial pain and jaw function might lower the disease burden for these patients as this could lead to conservative treatment being initiated at an earlier stage. As MBD such as anxiety and depression have an impact on TMD and the risk for chronic systemic diseases, mental health factors might also be important in patient-selection, together with timing of the treatment and better understanding of disease relapses for both odontological and medical caregivers [25–32].

The markedly increased odds of multiple surgical interventions in some of the diagnostic groups, such as inflammatory polyarthritis where no curative treatment exists, needs to be addressed. The repeated surgical treatments in these patient groups might not reflect failure of surgical treatment but rather reflect relapses in the systemic disease that causes increased TMJ symptoms and the need of new surgical interventions. For these patients, all available treatments, conservative or surgical, aim at symptom reduction and minimizing joint destruction, which in most cases leads to life-long treatments and in severe cases repeated surgery. It is imperative to understand that for these patients, prevention of repeated surgical interventions might not be possible.

The distribution of gender in patients suffering from TMD has been recognized for a long time, and women are reported to be 2–8 times more likely to develop TMD compared to men [13]. Our study had a male-female ratio of 1:2.7 in the entire TMD case group, and the gender difference displays a ladder pattern like the one found for different diagnostic groups. The NS group had a ratio of 1:2.6, whereas the ST1 and ST2 groups had ratios of 1:3.3 and 1:5.3, respectively. Studies have found women to be more healthcare seeking than men, which might partially explain these findings [33, 34]. Moreover, many of the diagnoses with high probability of developing TMD found in this study are more common among women, such as RA, juvenile idiopathic arthritis, systemic sclerosis, and osteoporosis [8, 35–38]. If these diseases are true causal risk factors, it is possible that these predominantly female diagnoses, of which some are quite common, also drive the male-female ratio within the TMD patients.

In Fig 2 the ORs are markedly decreasing over age in the categories arthropathies (M00-M25), systemic connective tissue disorders (M30-M36) and other disorders of the musculoskeletal system and connective tissue (M95-M99). A recent study on the impact of the association between rheumatoid arthritis (RA) antibodies, age and gender showed that certain antibodies were strongly associated to higher or lower age, where anti-CCP was associated with lower age and IgA-RF with high age of RA onset [39]. It is plausible that expression of different auto antibodies might also predict the onset of incident TMD. For some diagnoses such as systemic lupus erythematosus (SLE) the peak of incidence occurs during the reproductive years, which might partially explain the decrease of odds over time. Moreover, there is a pattern of higher frequencies of females than males in the pre-pubertal and child-bearing ages diagnosed with SLE [40]. Considering the gender differences within the study population and TMD patient group, the male-female ratio might further drive this peak of odds during the reproductive years. The opposite pattern is seen in Osteopathies and chondropathies (M80-M94) where the predominant diagnoses are disorders of bone density and structure (M80-M85). In this category the odds instead seem to increase with age, which might not be surprising as these diseases are strongly associated with higher age, in particular in women over 55 years of age and men over 65 years of age [41]. Although RA, SLE and osteoporosis do not account for the entire diagnostic categories, the difference in odds over age may partially be explained by the different pathophysiological progressions that are associated with each category of disease and may also in other study settings further inquire on the specific association to the development of TMD.

The strengths of this study are many, as it uses nationwide registry data that has been prospectively collected since 1964, using high quality registries with high validity and reliance [14–16]. The large number of study subjects offer possibilities of encountering associations even in rare conditions and the results of this study surely will contribute to the future knowledge and research regarding possible risk factors for the development of TMD. However, the results of this study should be interpreted in the light of some limitations, accompanying the design of registry-based studies. Whereas registries include exact and comprehensive sociodemographic data and other variables including ICD codes, the identification of TMD patients poses some challenges in registry research. The most apparent limitation is that TMD patients that are diagnosed within the hospital system but subsequently treated conservatively outside of it, comprise a small part of the entire TMD population, as most of these patients receive their diagnosis and conservative treatment within the dental care system and are therefore not registered in the NPR. On the other hand, patients surgically treated for TMD are very likely to be found within NPR, as TMJ surgery is always conducted within the Swedish health care system and registered in the NPR. It is therefore important to address that the conclusions drawn on the NS group is limited to a subset of patients that receive a TMD diagnosis within the hospital care system and thereafter undergo conservative treatment outside the hospital. Measurement bias associated with studies using electronic medical records has been mentioned earlier under possible bias but should be mentioned as a possible limitation even though it should be directed towards the null. Furthermore, in spite using incident cases for this study, they were incident within the boundaries of the health care system and NPR, and during 1998–2016. Therefore, there might be a subset of cases that have had TMD since before, causing the temporal relationship with MSD exposure to be uncertain. Using the ICD-10 code K07.6 for TMD can be suboptimal and the use of DC/TMD should be preferred, however the NPR does not hold information on findings from DC/TMD screenings and therefore the available data is on K07.6 alone. Also, a TMD diagnosis with no subsequent surgical treatment might not be equal to the patient suffering from myofascial pain and/or dysfunction, as there may be many reasons for the patient to not undergo surgery, such as fittingness to be anesthetized, anatomical difficulties or the patient’s unwillingness to endure surgical treatment. Another concern that must be mentioned is the disease evolution of different MSDs which might have effect on the development of TMD. This study does not investigate the effect of MSD relapse where the subjects can present with different levels of intensity of symptoms and subsequent treatment. Repeated relapses, certain treatment methods and the gradual deterioration might very well have a large impact on the development of subsequent TMD, through both biological and psychological pathways. These important matters, however, cannot be answered within the frameworks of this study design. Residual confounding that cannot be controlled for is always associated with any observational study but may need to be mentioned and considered when drawing conclusions on the results of this study.

Nevertheless, our findings strongly emphasize the role of MSDs in the development of TMD. Moreover, the advance of symptoms treated in different parts of the dental and health care system highlights the importance of using this knowledge in a multi-disciplinary clinical decision making to identify the patients who could benefit from surgical intervention. It is also evident that these patient groups are extremely vulnerable with a substantial amount of musculoskeletal comorbidities and a high disease burden. The findings shed further light on the need for research on disease-specific associations to identify possible causal pathways and underlying biological mechanisms.

## Conclusions

MSD comorbidities have a strong association to TMD, in particular TMD requiring surgical intervention. The results, which are in line with earlier findings, present new population-based evidence of a possible causal relationship between MSD and TMD, even after adjusting for known confounders. The implications of identifying potential risk factors associated with TMD development are important in future clinical management both in dental and medical care. In a longer perspective, these results may form the basis for more evidence-based selection of the patients who might be at higher risk for surgical treatment, and perhaps aid in the selection of potential candidates for surgery. Optimistically these findings may cause both physicians and dentists to be more aware of this possible causal link, causing attentiveness to early signs of orofacial pain in patients with MSD and increasing the chance of early referral and initiated conservative treatment–thereby bridging the gap between the dental and health care system.
